# Crossing lines: a multidisciplinary framework for assessing connectivity of hammerhead sharks across jurisdictional boundaries

**DOI:** 10.1038/srep46061

**Published:** 2017-04-21

**Authors:** A. Chin, C. A. Simpfendorfer, W. T. White, G. J. Johnson, R. B. McAuley, M. R. Heupel

**Affiliations:** 1 Centre for Sustainable Tropical Fisheries and Aquaculture, James Cook University, Townsville, Australia; 2 Australian Institute of Marine Science, Townsville, Australia; 3 Commonwealth Scientific and Industrial Research Organisation, Hobart, Australia; 4 Northern Territory Department of Primary Industries and Fisheries, Darwin, Australia; 5 Western Australian Department of Fisheries, Perth, Australia.

## Abstract

Conservation and management of migratory species can be complex and challenging. International agreements such as the Convention on Migratory Species (CMS) provide policy frameworks, but assessments and management can be hampered by lack of data and tractable mechanisms to integrate disparate datasets. An assessment of scalloped (*Sphyrna lewini*) and great (*Sphyrna mokarran*) hammerhead population structure and connectivity across northern Australia, Indonesia and Papua New Guinea (PNG) was conducted to inform management responses to CMS and Convention on International Trade in Endangered Species listings of these species. An Integrated Assessment Framework (IAF) was devised to systematically incorporate data across jurisdictions and create a regional synopsis, and amalgamated a suite of data from the Australasian region. Scalloped hammerhead populations are segregated by sex and size, with Australian populations dominated by juveniles and small adult males, while Indonesian and PNG populations included large adult females. The IAF process introduced genetic and tagging data to produce conceptual models of stock structure and movement. Several hypotheses were produced to explain stock structure and movement patterns, but more data are needed to identify the most likely hypothesis. This study demonstrates a process for assessing migratory species connectivity and highlights priority areas for hammerhead management and research.

The conservation and management of migratory species presents a complex suite of challenges. Highly migratory species encounter a wide range of natural and anthropogenic pressures during their movements, and may cross numerous jurisdictional boundaries which further complicate management efforts^1^,^2^. Conservation and management problems are magnified for marine species which may be data poor, can migrate thousands of kilometres and exhibit complex movement and migratory patterns that change in space and time^1^,^3^,^4^. Management of mobile marine species is also hindered by difficulties in accessing individuals for research and monitoring, and complexities in managing distant water, open ocean fisheries^5^. Furthermore, highly migratory marine species may also occur within ‘high seas’ areas beyond national jurisdictions^5^,^6^. Collectively, research, conservation and management are difficult to successfully implement for these species which occur in these expansive, remote oceanic regions^6^,^7^,^8^.

Pressures and impacts on migratory marine species are well documented. Fishing is the primary pressure with numerous studies documenting impacts on species such as tuna, billfishes and sharks^9^,^10^,^11^,^12^, as well as bycatch species such as seabirds, marine mammals, turtles and non-target sharks and rays^1^,^13^,^14^,^15^. Migratory marine species are also susceptible to impacts from global environmental change where changes in oceanic upwelling patterns, water chemistry, biological productivity, current patterns and phenology may have severe consequences^16^,^17^,^18^,^19^. The protection of migratory sharks has been of increasing conservation concern due to a combination of their life history traits that limit their reproductive output and rebound potential, high fisheries mortality, and documented population declines^11^,^15^,^20^. Indeed, conservation assessments have found that a relatively high proportion of migratory sharks are amongst those sharks and rays of highest risk from fishing^21^. These concerns have led to regional fishery management organisations implementing bans on the retention of some species such as the oceanic whitetip shark (*Carcharhinus longimanus*), silky shark (*C. falciformis*) and hammerhead (*Sphyrna* spp.) sharks^11^,^22^, and their inclusion in the Convention on International Trade in Endangered Species (CITES; Appendix II) and the Convention on Migratory Species (CMS; Appendix II).

Hammerhead sharks are increasingly recognised as species of conservation concern^11^,^15^,^23^. There are currently ten recognised species that can be grouped based on the maximum lengths they attain: large species >1.5 m (*Sphyrna gilberti, S. lewini, S. mokarran, S. zygaena* and *Eusphyra blochii*) and smaller species, <1.5 m (*S. corona, S. couardi, S. media, S. tiburo* and *S. tudes*). Conservation concerns and management actions have focused on the group of larger more widely distributed species. Three of these species (great hammerhead, *S. mokarran*; scalloped hammerhead, *S. lewini* and winghead shark, *E. blochii*) occur in northern Australia and the Indo-Australian archipelago^24^. A fourth species (smooth hammerhead *S. zygaena*) has a more temperate distribution in Australian waters, but does sometimes occur in tropical waters^25^,^26^. Within this region hammerhead sharks are commonly taken in fisheries as both target and incidental catch for both their flesh and fins^26^,^27^.

Hammerhead conservation assessment findings vary between the different hammerhead species and locations, reflecting their wide distribution and complex movement. Based on IUCN Red List assessments the winghead shark is currently considered Near Threatened globally (Simpfendorfer 2003) but Least Concern in Australian waters (Table 1). However, it is worth noting this species has a more patchy distribution and is poorly studied. In contrast, scalloped and great hammerheads are more commonly encountered and considered at greater risk of extinction than the other two large species (the smooth hammerhead and the Carolina hammerhead *S. gilberti*). Both the scalloped and great hammerhead are assessed as globally Endangered^28^,^29^ and as Vulnerable and Endangered in Oceania, respectively (Table 1). Meanwhile, the smooth hammerhead is listed as globally Vulnerable. In addition to IUCN Red List assessments, recent listings on CITES Appendix II (all three species) in 2014 and the CMS Appendix II in 2015 (scalloped and great hammerhead) highlight global concerns over their status and the need for conservation action. Listing on CITES Appendix II and CMS Appendix II requires nations that share species to work together to manage shared populations. Meeting these obligations requires an assessment of hammerhead population status, stock structure and connectivity across different range states and jurisdictions. Assessing stock structure and connectivity are particularly important for migratory species because this information can help identify the nations and agencies that need to work together to assess and manage these species, and identify the different risks and potential management responses in each jurisdiction.

Within the Australasian region, the great hammerhead, scalloped hammerhead and winghead shark occur across northern Australia, Indonesia, Papua New Guinea (PNG), and the scalloped hammerhead has also been documented in the Solomon Islands. In Australia, the species have overlapping ranges across Western Australia to New South Wales, in habitats ranging from the continental shelf to shallow inshore waters^24^. Their distributions within Indonesia and PNG are not well documented, but are likely widely distributed across these countries. Scalloped hammerheads also occur in open ocean habitats and are known to make long distance migrations^30^,^31^.

Conservation and management efforts for the scalloped and great hammerhead sharks are complicated by their ability to move large distances, their wide distribution across different jurisdictions, and data gaps on movement and occurrence. Typically, assessing the status of, threats to, and management options for migratory species presents a complex suite of problems that may require large amounts of data. For mobile marine species, required data include the species’ distribution and relative abundance at all life history stages across its range, movement and migration patterns, fisheries catch and effort, as well as the species’ responses to environmental factors and how these responses affect exposure to pressures^1^,^4^,^32^. These factors also need to be considered alongside habitat use and life history traits which affect sensitivity and resilience to pressures. For example, slow growth and long lifespans of large bodied pelagic sharks increases their vulnerability to fishing threats^15^, while sharks and rays with strict habitat requirements are more vulnerable to climate change^33^. The large number of variables and interactions involved presents a challenge that requires a systematic approach to interpret the data. Numerous approaches have been developed to assess threats and inform management amongst complex social-ecological systems with a large number of variables^34^,^35^, however the majority of these approaches are a form of Integrated Assessment where disparate data are collated and systematically processed to produce a single, synthesis assessment.

In simple terms, an Integrated Assessment is a process of aggregating diverse datasets into a composite understanding. Rotmans & VanAsselt^36^ (page 327) defined an Integrated Assessment as *“an interdisciplinary and participatory process of combining, interpreting and communicating knowledge from diverse scientific disciplines to allow a better understanding of complex phenomena*”. An Integrated Assessment has two main characteristics. It (1) constructs new knowledge and value by creating a composite understanding from different disciplinary research, and (2) is designed to provide decision makers with useful information^36^. Integrated Assessments range from qualitative assessments based on expert elicitation^37^ to quantitative assessments which include fully parametrised modelling approaches such as used in catchment assessment^34^. These integrative assessments can also be scaled according to the amount of data available, applying complex quantitative modelling approaches in data rich situations and using semi-quantitative conceptual approaches where data are scarcer^38^.

The flexibility of Integrated Assessment approaches make them ideally suited to assessing migratory marine species where data are patchy and highly variable. The present study demonstrates the application of a simple Integrative Assessment Framework to identify and integrate disparate datasets across jurisdictions and scientific disciplines. The framework presents composite data and conceptual models that provide a regional scale synthesis of the stock structure and potential migration of scalloped and great hammerhead sharks throughout the Indo-Australasian region, specifically Indonesia, Northern Australia, and Papua New Guinea. These countries were selected as they contain active fisheries that interact with these species, have catch and/or occurrence data available regarding these species, and also have programs to assess and improve the management of shark fisheries within their waters.

## Methods

Constructing a regional scale assessment of migratory species requires a systematic, collaborative and integrative approach as data on these species are often disparate, limited, and spread across different agencies. Following established methods^36^, this study developed a simple Integrated Assessment Framework (IAF) to provide a clear, systematic and repeatable method of identifying, collating, integrating and analysing the available data about hammerheads in the Australasian region. The IAF followed seven sequential steps loosely modelled on existing assessment frameworks for assessing climate change and environmental impact^33^, and aligned with the three main components of an IAF: (1) context; (2) process and (3) method^39^.

The first step **(1)** ***Definition*** clearly defined the scope and scale of the assessment. This process identifies the actors and stakeholders needed to help formulate the questions, development of specific research questions, identifying the datasets needed to resolve them, and key assumptions. This step can also clarify the questions and issues that are beyond the scope of the study. Step **(2)** ***Acquisition*** is a process step to conduct a data audit and collate the available data. *Acquisition* was facilitated by an expert workshop in February 2015 to assess the conservation status of Australian sharks and rays using IUCN Red List criteria. The workshop brought together experts from across the region, and this process and follow-up engagement helped identify available hammerhead species data. Once datasets were collected, data were standardised and checked. Data standardisation involved converting spatial data into similar coordinate systems and processing data to ensure the datasets used included the required information (length, sex, location). The *Acquisition* step also included standardising length measurements between studies (using published length conversions between fork length (FL), total length (TL) and stretched total length (STL) as well as regression relationships between these measurements). TL was used as the standard length measurement as it was the common length metric used across all datasets and is commonly used for shark studies^40^. Maturity stages for individual records were assigned using published size-at-maturity data from the nearest region^41^,^42^. Once data were processed, they were **(3)** ***Visualised*** to identify the main patterns. Visualisation entailed integrating datasets into a single representation using Geographic Information System ArcGIS, and integrating size and sex data into size and sex frequency histograms for each species within each jurisdiction. This process generated maps and graphs showing the distribution of hammerhead species, their size and sex characteristics throughout the assessment region, and size and sex structures between jurisdictions. Once spatial patterns and sex and size structures were visualised, the composite data were **(4)** ***Validated*** by qualitative inspection to identify outliers and unexpected and unexplained patterns. These phenomena were highlighted and the source data checked and verified with data contributors. Once the visualised data and patterns were validated, patterns were **(5)** ***Interpreted*** to identify the main trends in size and sex structure and potential for stock sharing across the assessment region, taking into account how differences in sampling design and effort could affect these interpretations. Interpretations were **(6)** ***Explored*** by examining the patterns in population size, sex structure, and distribution. A literature review, additional data about movement and migration of conspecifics or species analogues, genetic connectivity information and expert opinion were brought together to construct conceptual models of movement, connectivity and biogeography. These models integrated the disparate data types. Conceptual models attempted to explain the observed patterns and were used to develop hypotheses. Finally, models and hypotheses were **(7)** ***Qualitatively Assessed*** by categorising the level of support available for each conceptual model and by identifying what data would be needed to test each model. Models that had limited support were assessed as the least plausible models, while those with high support and high certainty were considered the most plausible.

Categories describe the amount of support from the available data were as follows:

- Limited support: model is not believed to represent real world case; limited support (no data available or data contradicts the model) for the model.
- Moderate support: model could support real world case; there are several data sources that provide direct or circumstantial evidence to support the model.
- Highly supported: model likely to represent real world case; high congruence between multiple datasets providing direct and/or circumstantial evidence to support the model.

## Results

### Definition, acquisition, visualisation and validation

**Figure 1:**
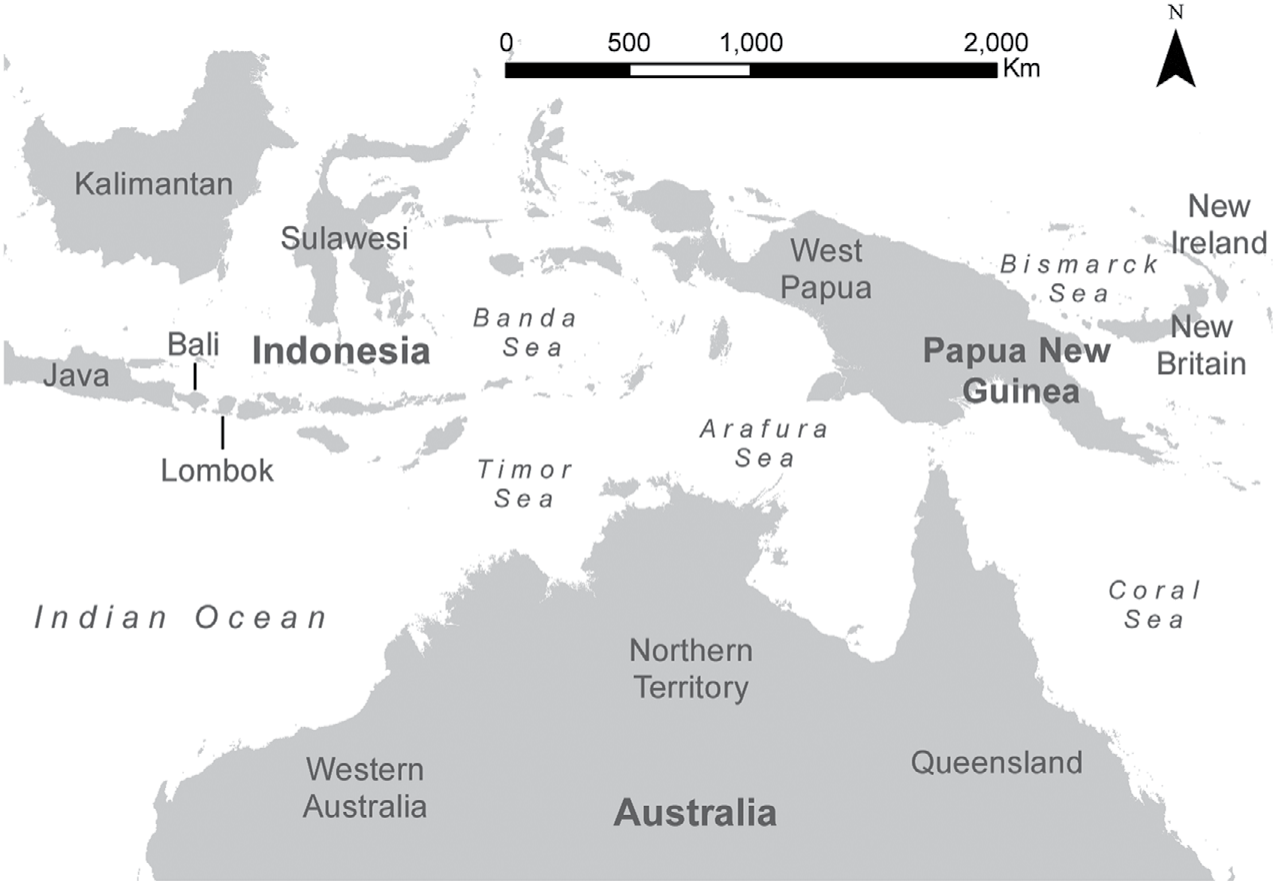
Locality map showing the scope of the asessment region across Australasia and Indonesia. The map shows the countries and major regional features discussed during the assessment. Figure created using ArcGIS 10.2.1.

The context and specific requirements of the project were clearly **defined** through involvement of fisheries managers and stakeholders. These representatives provided guidance regarding the type of information needed to assist the Australian Government in responding to CITES and CMS listings of hammerhead species, and inform internal policy regarding management of these species. The geographic area of interest included Western Australia, the Northern Territory, Queensland, the Torres Strait, PNG and southern Indonesia, henceforth referred to as the Assessment Region (Fig. 1). The aims and scope revealed a need to source occurrence, distribution and fishery data from across the Assessment Region to compile a regional scale synthesis of hammerhead catches and fisheries interactions. For the present study, the specific aim was to *provide a preliminary description of the size and sex structure of great and scalloped hammerhead shark populations in Northern Australia, Indonesia and Papua New Guinea, and the extent to which populations are shared between these jurisdictions*.

The **acquisition** phase involved securing successful collaborations and agreements that brought together nine separate datasets from Australia, Indonesia and PNG from a range of government agencies and independent research institutions (Table 2).

The acquisition process produced 6,938 records between the datasets for the two species: 5,182 records for scalloped hammerheads (Australian data n = 4,213; Indonesia n = 706 and PNG n = 263); and 1,756 records for great hammerheads but noting that over 99% of these records were from Australia (Australian data n = 1,745; Indonesia n = 11 and PNG n = 0).

Fisheries catch and observer data provided the majority of location data, and were also the only datasets with robust size and sex data. Spatial data indicating hammerhead occurrence and distribution were available for Western Australia, the Northern Territory, Queensland and Papua New Guinea, but were not available for Indonesia. Unfortunately, data from Indonesia were limited to surveys of fish markets in southern Indonesia on Java, Bali and Lombok (Fig. 1), including six markets: Cilacap (Central Java), Kedonganan (Bali), Muara Angke (Java, Jakarta), Muara Baru (Java, Jakarta), Pelabuhanratu (West Java) and Tanjung Luar (Lombok)^43^. While these markets are the largest such markets in the region, it was not possible to determine the catch location of the samples and as such, precise catch-location data could not be determined. However it is likely that some of the landed catches were sourced from designated fishing areas in Australian waters that are frequently accessed by Indonesian fishermen under a formal Memorandum of Understanding between Australia and Indonesia. Similarly, little useable spatial distribution data could be derived from the Queensland shark control dataset as the gear is set at a small number of permanent locations, and catch data are confounded by changes gear type and deployment.

**Figure 2:**
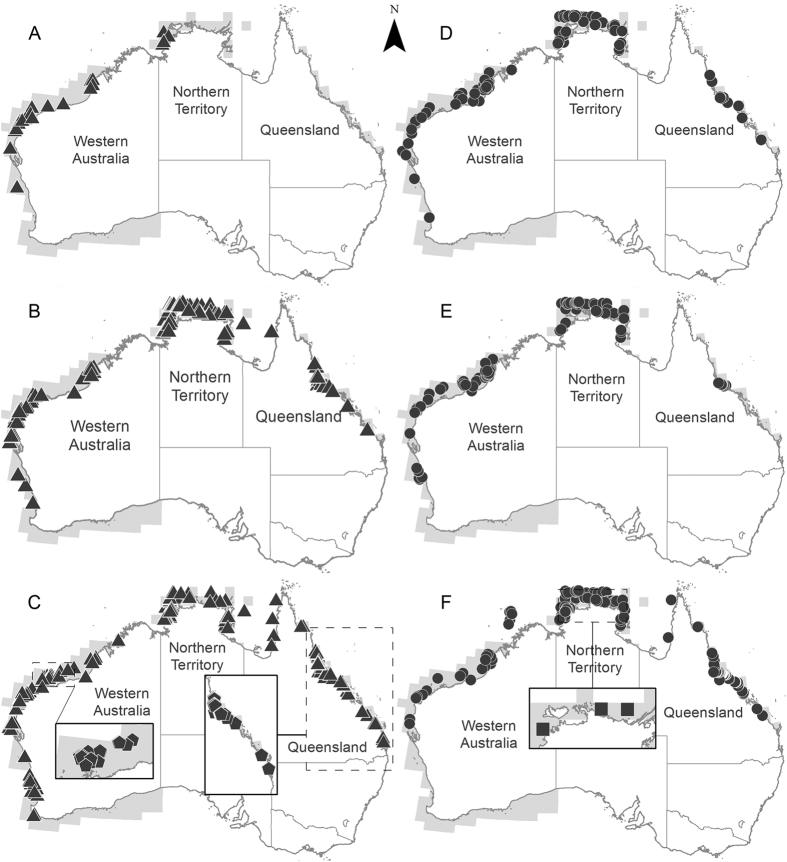
Indicative distribution of: (**A**–**C**) scalloped hammerhed (triangles) and (**D**–**F**) great hammerhed (circles) sharks for sex and size categories from sampled locations. (**A**,**D**) adult females, (**B**,**E**) adult males, (**C**,**F**) immature and neonate individuals of both sexes. Insets show indicative distribution of neonates. Grey shading denotes spatial grids where fishing and sampling effort occurred. Note: more detailed information on fishing effort is not available due to confidentiality provisions of data-sharing agreements and fisheries monitoring programs. Data for scalloped hammerheads from PNG presented in Fig. 4. Spatial information for Indonesia was not available. Figure created using ArcGIS 10.2.1.

**Figure 3:**
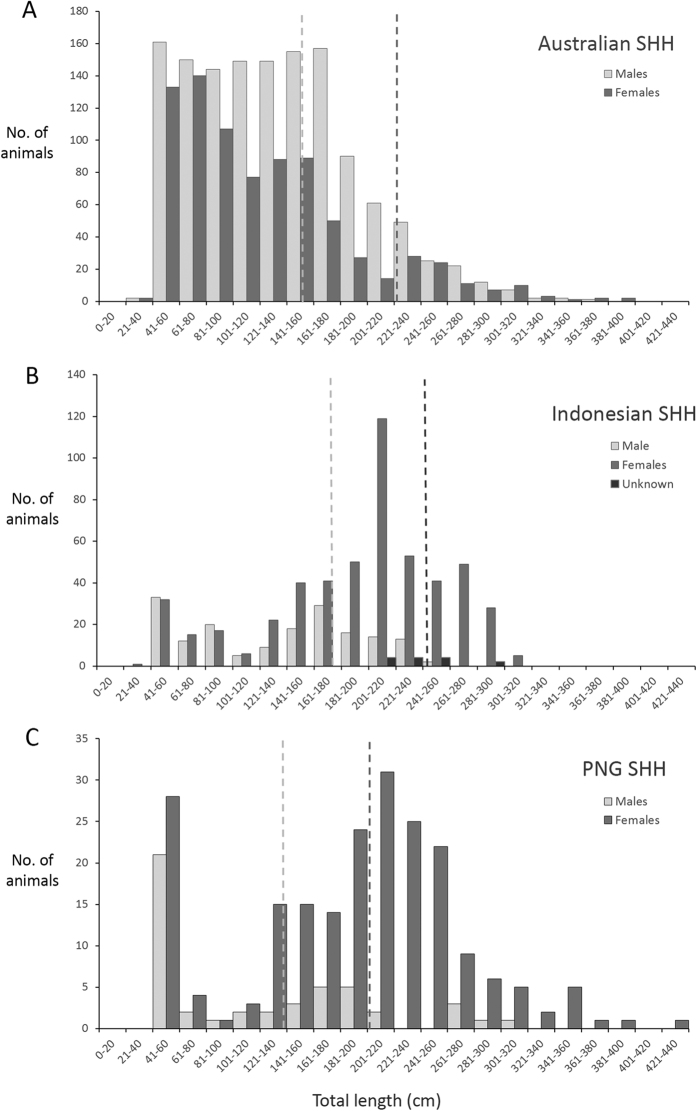
Size frequency distributions of scalloped hammerheads recorded in fisheres catches in Australia (**A**), Indonesia (**B**) and Papua New Guinea (**C**) showing the low numbers of adult females in Australian waters but occurrence in Indonesia and Papua New Guinea. Dotted lines indicate size at maturity for males (light grey) and females (dark grey).

**Figure 4:**
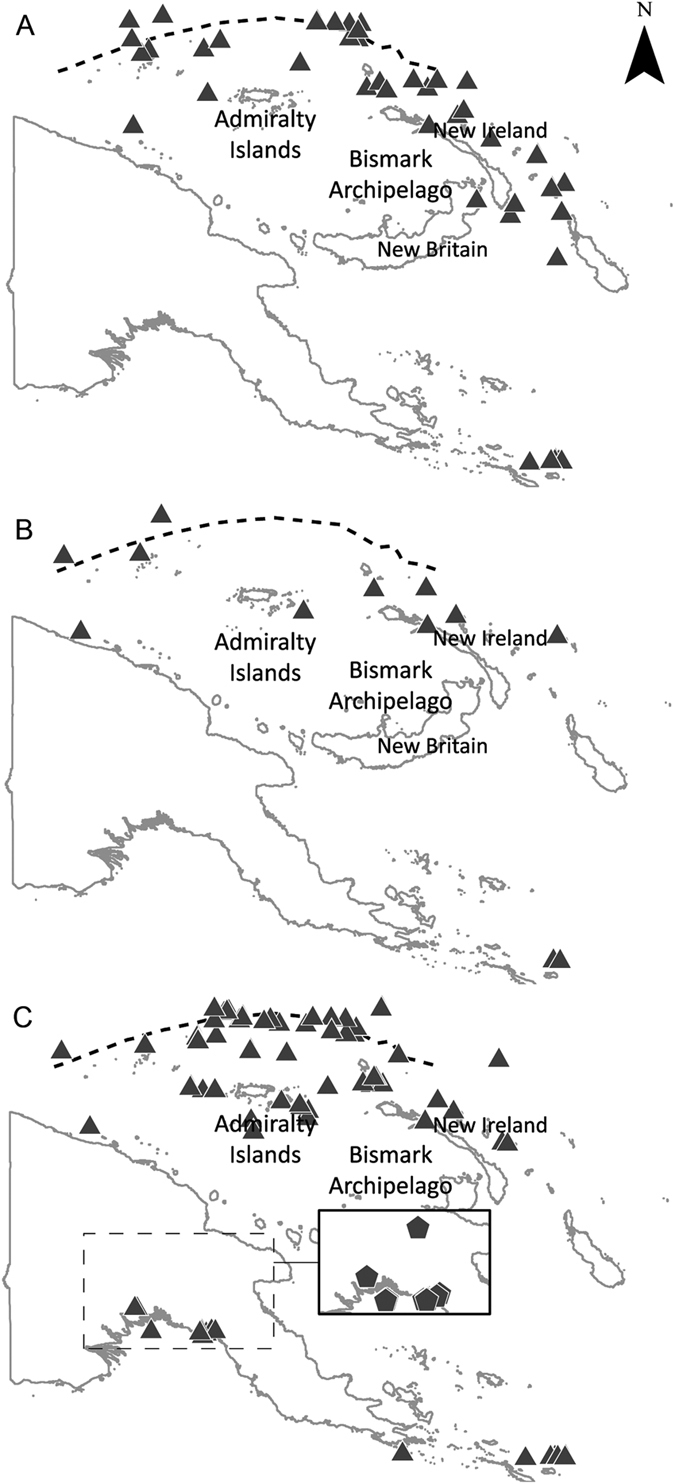
Distribution of scalloped hammerheds in Papua New Guinea. (**A**) adult females, (**B**) adult males, (**C**) immature and neonate individuals of both sexes. Insets denote neonates (pentagons). Dashed line represents the edge of the continental shelf – North Bismark Plate. Figure created using ArcGIS 10.2.1.

The **visualisation** step produced distribution maps of each species’ occurrence in Australia as well as indicative fishing effort (Fig. 2). Size frequency histograms were also produced for scalloped hammerheads from each dataset (all Australian data combined, Indonesia and PNG) (Fig. 3). Size frequency data were not plotted for great hammerheads as the lack of catches of great hammerhead sharks in Indonesia and PNG prevented comparisons of size structures for this species. Spatial data for hammerhead catches in PNG were also mapped (Fig. 4), however detailed information on fishing effort was not available. Spatial data files (Google Earth™ KMZ files) were produced from all mapped data so that collaborators and dataset owners could use to visualise and manipulate the data. Collaborators were invited to **validate** these data outputs to ensure they accurately represented fisheries catches and occurrence records. During this validation process, records where spatial data were obviously flawed (e.g. catch locations on land or in the northern hemisphere) and could not be corrected were discarded from the dataset. Distribution maps were also generated from data in national databases holding community sourced citizen science information. However, when the data were mapped, hundreds of data points were generated in locations in temperate waters (e.g. the Southern Ocean) where scalloped or great hammerheads do not commonly occur. Unfortunately, efforts to identify and remove suspect data from these datasets were unsuccessful in resolving the erroneous spatial data. The dataset contained over 2,112 records (1,820 scalloped hammerhead; 292 great hammerhead) pooled from a large number of organisations and individuals. Suspect datasets were identified based on their date and sources known to have had misidentification issues, but subsequent mapping efforts where suspect datasets were sequentially removed failed to correct the erroneous results. Subsequently, it was decided to exclude the citizen science data from the analysis. The eight remaining datasets (Table 2) included 4,826 data records; 3,362 for scalloped hammerhead sharks and 1,464 for great hammerhead sharks. These data proved invaluable to illustrating population structure. Once data were validated, the project team reviewed the regional trends and emerging patterns to **interpret** the aggregate data, and to identify the main patterns.

### Interpretation: size and sex population structures

The main emergent pattern from the included datasets was that species occurrence and population structure were markedly different across the region. Scalloped hammerheads occurred widely across northern Australia (n = 2,393), Indonesia (n = 706) and PNG (n = 263), however great hammerheads were much more commonly reported from Australian waters than other countries (n = 1,453) with very few records from Indonesia (n = 11) and none from PNG (n = 0) (Fig. 2). Population size and sex structure for scalloped hammerheads also differed across the assessment region. Australian populations were dominated by neonates and juveniles, with some adult males but very few adult females (Fig. 3). In contrast, adult females comprised a larger proportion of adults from Indonesia and PNG (Fig. 3). While the lack of great hammerhead data from Indonesia and PNG prevented comparisons of size and sex structure, the available data show that Australian great hammerhead populations are dominated by neonates and juveniles, with few adult males recorded. Similar to records for scalloped hammerheads, very few adult females were reported. The distribution of adult female scalloped hammerheads was of particular interest given their scarcity in Australian datasets, and their demographic importance to reproduction and subsequent population replenishment. While market surveys showed that adult female scalloped hammerheads occurred in Indonesian markets, the only spatial data showing large numbers of adult females was from PNG where records indicated that large females were caught along the Bismarck Archipelago, mainly in eastern New Britain and northern New Ireland, and scattered around the Admiralty Islands (Fig. 4).

During the interpretation process, it was clear that the spatial patterns of hammerhead occurrence could be biased by fishing and observer effort, i.e. hammerheads are not reported from areas where fishing and/or reporting are limited. In Australia, sampling effort covered large continuous stretches of coastal waters in Western Australia (WA) and the Northern Territory (NT), and some of the Queensland (Qld) east coast. However, fishing effort was limited in offshore waters, and in coastal waters along northwest Queensland and the southern Gulf of Carpentaria (Fig. 2A). While comparable fishing effort data were not available for PNG, anecdotal reports suggest that fishing effort is concentrated offshore and thus, there was limited sampling of coastal regions. While this type of bias is common in fisheries datasets, the ramifications of these potential biases need to be carefully considered (see Discussion).

### Exploration and assessment: conceptual model formulation and assessment

Patterns in scalloped hammerhead distribution and population structure were compared between the sub-regions, and hypotheses developed to conceptually explain the observed patterns. Each hypothesis was qualitatively assessed for level of support based on available literature and expert knowledge of the species biology and connectivity. Unfortunately, the lack of data for great hammerheads outside Australian waters precluded exploration of migration and connectivity patterns for this species, so hypotheses and models are only presented for the scalloped hammerhead shark.

Currently a range of fragmentary information exists regarding the stock structure of scalloped hammerheads. This information is summarised below.

**Figure 5:**
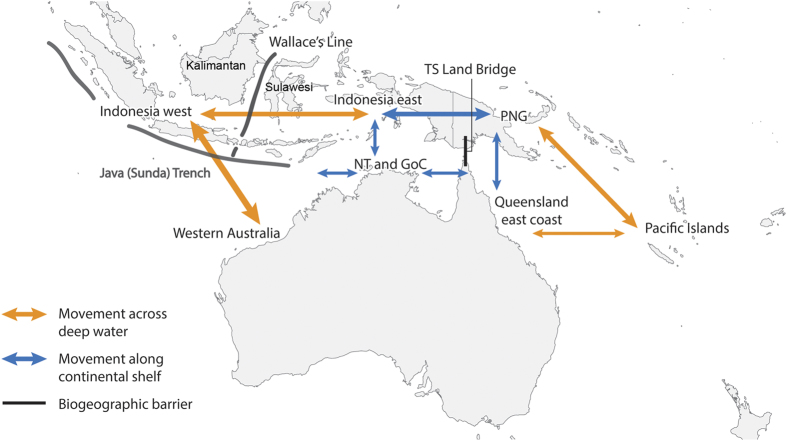
Conceptual population structure model of scalloped hammerhead sharks in Australasia. TS indicates the Torres Strait land bridge, GoC is the Gulf of Carpentaria, NT is the Northern Territory, Figure created using ArcGIS 10.2.1 and biogeographic features added using Adobe Photoshop CC2015.

1. Size and sex structure data indicate that few adult females (and especially pregnant females) occur in northern Australia, but are regularly caught in Indonesia and PNG. Considering that hammerheads sharks in other regions move to shallow nursery areas to give birth^31^,^44^,^45^, this population structure suggests that a proportion adult females may migrate from Australia to Indonesia and PNG, but return to give birth to their young in nursery areas in coastal areas of northern Australia^46^,^47^.
2. There is genetic evidence of mixing between Australian and Indonesian animals^48^. Current genetic analysis only provides evidence of a connection on evolutionary time scales (although it does not discount connections at shorter time scales). This evidence does not discount the possibility that adult female scalloped hammerheads regularly migrate north from Australian into Indonesia and PNG.
3. There are a number of identified biogeographic barriers in the region (Fig. 5) that may be important for structuring the population:
  - Torres Strait Land Bridge. This may form a barrier between stocks on the east coast of Queensland and the rest of northern Australia as reported for other species of coastal sharks (e.g. common blacktip shark, *Carcharhinus limbatus*) and coastal pelagic teleosts (e.g. grey mackerel, *Scomberomorus semifasciatus*)^49^.
  - Deepwater between Australia and Indonesia (Java Trench) (Fig. 5). This may form a barrier to regular movement between Western Australia and the Northern Territory north into Indonesia. However, genetic similarities between Australian and Indonesian specimens mean this may not be the case^50^.
  - Wallace Line. This well-known biogeographic barrier running through Indonesia (between Bali and Lombok in the south, and running north between Kalimantan and Sulawesi) is a result of deep water that may form a barrier to movement between eastern and western Indonesia (Fig. 5). This may mean individuals west of the Wallace Line have less connection with northern Australia compared to those to the east because that region shares a continental shelf with northern Australia.
4. Genetic evidence from other parts of the world suggest females have a tendency to remain associated with particular stretches of continental shelf and display natal philopatry (i.e. return to the nursery area in which they were born to give birth)^51^,^52^. If applicable to the northern Australian population it would preclude a strong connection to western Indonesia. However, the shared continental shelf with New Guinea and eastern margin of the Banda Sea (Indonesia) would not preclude this connection.
5. Genetic evidence from other parts of the range indicates males move over larger distances and have less population structure than females^53^. This suggests that genetic markers of female gene flow (e.g. mitochondrial DNA) may provide different results than genetic markers that are derived from genetic material from both sexes (microsatellites, single nucleotide polymorphisms [SNPs]).
6. Evidence from tagging and telemetry studies show that adult scalloped hammerheads can travel long distances, including across open oceans^30^. If these movement patterns exist in Australasian populations this would allow for movement between northern Australia, Indonesia, PNG and the broader Pacific and Indian Oceans.

Exploring the patterns arising from the scalloped hammerhead data combined with existing information yielded four conceptual models that integrated data on biogeography, genetic structure and movement to explain the observed patterns of distribution and population structure (Table 3, Fig. 5).

These four models incorporated movement across shallow continental shelf habitats and movements across deep waters. The models were also not exclusive, with some being extensions of others. The model with the highest current support is the panmictic population model (Model 1) which described continental shelf movement as well as movements across deep water to Indonesia and PNG. However, the continental scale movement (Model 3) and east-west divide and continental scale movement (Model 4) are also plausible, and are not incongruent with Model 1.

## Discussion

Management and conservation of migratory species is often complicated by incomplete knowledge about species movement patterns and mixing levels amongst populations; by dynamic and diverse movement patterns and driving forces; and varying management and conservation strategies across jurisdictions^5^,^54^. Using hammerheads as a case study, the IAF addressed this complexity by providing a structured approach to collating and assessing the available data, and producing conceptual models of population structuring and connectivity. Examining the available data on distribution of size and sex stages, life history parameters and genetic relatedness within a biogeographical context produced a likely scenario of connectivity within Australasia with no apparent boundaries to movement. This hypothesis can now be tested through targeted research into the movement (e.g. tagging and telemetry) and genetic relatedness of individuals within the region. Completion of the IAF thus provides a basis for future research and a roadmap for similar assessment of other migratory shark species.

The IAF process was greatly assisted by the existence of established inter-agency collaborations and professional networks that facilitated communication and data exchange. A second key factor was having access to accurate, species-specific catch data from fisheries agencies and monitoring programs. Despite hammerheads’ easily recognisable unique head shape, individual species can be difficult to differentiate and are often recorded simply as “hammerhead” in catch records. The similarity between species has led to limited or confused data on the status and trends of hammerhead populations worldwide^55^,^56^. The overlapping distribution and migratory nature of hammerhead species also make accurate species identification even more important to defining populations. Furthermore, the various hammerhead species have different biological characteristics and occupy different ecological niches despite their overlapping distributions^27^, meaning that generalisations cannot be made across species^57^ and management requires species-specific data on distribution, movement and connectivity. In this instance, reliable observer programs and fisheries data were invaluable in addressing the issues of misidentification or combined taxonomic groupings which often complicate shark fishery management^58^,^59^. The use of trained observers in fisheries can resolve species misidentification issues^60^, and observers can also photograph confusing specimens for verification by experts^58^. Observers can also collect genetic samples to further validate species identification and for use in detailed genetic analysis^59^. Accurate species identification, location and biological data are crucial to complete assessments migratory shark species connectivity and management, and thus observer programs will be a critical component of future management programs for migratory hammerhead species and other sharks and fishes. Meanwhile, species misidentification is likely the key issue leading to the exclusion of citizen science data in this study, with these records probably being misidentifications between the great hammerhead and smooth hammerhead. It should also be noted that the lack of great hammerheads occurrences in PNG catch data and Indonesia fish market data probably reflect the real absence of this species from these catches. Both of these programs involved high level taxonomic expertise and photographic validation of observer data, and as such, misidentification of great hammerheads as scalloped hammerheads was considered unlikely.

These examples highlight the challenge of integrating datasets of differing rigour and quality into an assessment. A potential solution would be to develop a weighting system or a set of confidence criteria and categories to describe the reliability of each dataset. For example, datasets that include documented Quality Assurance and Quality Control (QA/QC) processes such as observer training and assessment, data verification and validation protocols, and program evaluation processes could be designated as High Confidence Datasets. Likewise, citizen science datasets that had documented QA/QC processes and used verified photographs of species to ensure correct identification records could also be considered as High Confidence Datasets. Indeed, citizen science photo-identification of whale sharks and mantas has been highly successful in tracking their population trends and movements^61^,^62^,^63^. Meanwhile, datasets without documented QA/QC or expert validation would be classified as Low Confidence Datasets. Explicitly describing the confidence of each dataset used in an IAF would help to define the limits of uncertainty in the assessment findings and models. In the present study, the eight datasets used were sourced from formal scientific research programs or fisheries observer programs with QA/QC procedures and thus, are considered as reliable, High Quality Datasets.

The collation of these high quality, species-specific data on hammerhead size, sex and location made it possible to hypothesise about hammerhead population structure in Australasia. However, the interpretation of these patterns are still unclear. The very low occurrence of large females in northern Australia coupled with large catch of adult females in Indonesia and PNG suggests a single population mixing across international boundaries. However, it is *also* possible that large females are present in Australian waters but were not detected from fisheries data, i.e. Australian fisheries do not operate in areas where adult females reside. For example, some pregnant females have been recorded, including in the Queensland Shark Control Program data^64^ (although there remain some concerns about identification of these animals). Visualisation of PNG data suggest large females may be attracted to specific habitat features such as ridges or steep drop-offs along the edge of continental shelves, areas that are not targeted by Australian shark fisheries. Targeted sampling of these habitat features in Australia may reveal similar female populations that are currently unknown. Similarly, the absence of great hammerhead sharks from PNG could reflect sampling bias and it is possible that targeted fishing in coastal regions of PNG could locate great hammerhead sharks. These examples highlight that the data used in this IAF were largely from fisheries activity and thus, are biased toward areas where target species occur. The use of different fishing gears in different locations may also affect the size ranges of the hammerhead sharks caught due to gear selectivity effects which are well documented in fisheries^65^,^66^. Consequently, some sub-regions and locations may be over-represented while others that are lightly fished are under-represented, and some animal size classes may actually be present but evade captured due to gear selectivity effects^66^. As such, more comprehensive data including fishery independent sampling using multiple gears, and perhaps targeted citizen science programs that use photographic verification, are likely to be needed to produce a more accurate and comprehensive account of hammerhead shark occurrence and connectivity across the region.

While catch data were vital to defining populations status, genetic analysis of sampled individuals were also critical to defining the extent of populations and their connectivity. Genetic analysis may also provide proxy information about movement patterns and connectivity where direct data from tagging and tracking are not available^67^. Previous research has revealed the highly conserved nature of shark DNA and the ability of a small number of individuals to produce population level connectivity^68^. Currently available data for scalloped hammerhead populations follows this pattern by indicating broad connectivity of populations within Australasia and the broader Pacific^48^,^53^. These results indicate at least some level of genetic mixing over broad spatial scales. However, advances in genetic techniques and analytical approaches are allowing increasingly higher resolution analyses to be conducted. These new approaches may provide population-level definition not possible with previous methods^69^,^70^. Therefore coordinated genetic tissue sampling and application of novel genetic techniques will be crucial to fully defining regional population connectivity.

The broad connectivity across the region indicated by genetic data, and large-scale movement capability indicated by tag-recapture data for these species from other regions^48^,^53^ reduce support for the hypotheses based on limited movement scenarios. Hypotheses related to continental shelf scale movements are possible, but given global the distribution of hammerhead species and their ability to cross open ocean areas^30^, seem unlikely. This leads to stronger support of a panmictic population model where high levels of connectivity occur and may be highly likely due to the close proximity of Australia, Indonesia and PNG. It is plausible that individuals could swim these distances, and also that adult females that mainly reside in Indonesia and PNG could routinely travel to northern Australia to give birth. However, the evolutionary time scale of the current genetic data limit the ability to draw conclusions on connectivity at shorter time scales, and while the panmictic population model is the most likely candidate, more data are needed to definitively state that this is the model that explains hammerhead population structure and connectivity in the region. Nevertheless, in the current situation where connectivity is not understood beyond broad genetic and distributional data we must assume a combined (panmictic) Australasian population. This assumption is the most sound and precautionary approach until further data can be collected to support or refute this hypothesis.

The proposed high level of regional connectivity has significant consequences for managing hammerhead shark populations. As nations develop policy responses to CITES and CMS listings, the connectivity of local populations and implications of national and international fishing pressure must be considered. To properly understand hammerhead population structure and connectivity across the region and beyond, targeted research is needed including high resolution population genetics, identifying movement patterns of individuals, and sampling of under-represented regions or key habitats. This includes the investigation of the connectivity of hammerhead populations between Australia and nations in the wider Indo-Pacific region where hammerheads are known to occur^44^. The IAF process could also be broadened to involve additional review and refinement. For example, the *Exploration* stage could introduce a process of external peer review or refinement to identify faults or omissions in conceptual models developed by the assessment group.

Although national plans have been or are being developed for managing hammerhead species under CITES and national threatened species laws, current and future plans should consider cross-jurisdictional connectivity and its management implications. This scenario is likely to exist in regions beyond Australasia and certainly applies to other highly migratory sharks. As such joint management arrangements must be a consideration to ensure effective management of migratory populations. The application of an IAF can and should be extended to other regions where hammerhead species occur (e.g. Western Central Pacific and Indian Oceans), and also applied to other at-risk migratory species (e.g. oceanic whitetip shark, *Carcharhinus longimanus*). Use of this framework has defined the extent of current knowledge, identified gaps and defined the critical questions around hammerhead shark status and management in Australasia. We suggest that this approach will be equally beneficial in other regions and for other species and provides a roadmap for directed science to support national and international policy and management of these species.

## Additional Information

**How to cite this article**: Chin, A. *et al*. Crossing lines: a multidisciplinary framework for assessing connectivity of hammerhead sharks across jurisdictional boundaries. *Sci. Rep.* **7**, 46061; doi: 10.1038/srep46061 (2017).

**Publisher's note:** Springer Nature remains neutral with regard to jurisdictional claims in published maps and institutional affiliations.

## Figures and Tables

**Table 1:** Conservation listings for hammerhead species under the International Union for the Conservation of Nature (IUCN) Red List, the Convention on International Trade in Endangered Species, and the Convention on Migratory Species.

	IUCN	CITES	CMS
Scalloped hammerhead shark	Endangered (global) Vulnerable (Oceania)	Appendix II (2014)	Appendix II (2015)
Great hammerhead shark	Endangered (global) Endangered (Oceania)	Appendix II (2014)	Appendix II (2015)
Smooth hammerhead shark	Vulnerable (global)	Appendix II (2014)	
Winghead shark	Near Threatened (global) Least concern (Australia)		

**Table 2:** Datasets collated and used showing jurisdictions and the types of data included.

Jurisdiction; data sources and data size (data sources in parentheses)	Dataset	Data types
Western Australia (WA Fisheries)	Fisheries observer data; fisheries logbook data; fishery independent research data	Species, size, sex, location
Northern Territory (NT Fisheries)	Fisheries observer data; fisheries logbook data; fishery independent research data	Species, size, sex, location
Queensland (James Cook University)	Fisheries observer data; fishery independent research data	Species, size, sex, location
Papua New Guinea (PNG National Fisheries Authority)	Fisheries observer data; fisheries logbook data and fishery independent research data	Species, size, sex, location
Indonesia (CSIRO*, RCFMC^#^)	Fish market surveys from independent research data	Species, size, sex
Queensland (Qld Fisheries)	Shark control program catch records	Species, size, sex, location
Western Australia, Queensland, Northern territory (Australian Institute of Marine Science)	Baited remote underwater video surveys	Species, location
Australia wide	Citizen science data	Species, location
Western Australia (WA Fisheries), Northern Territory (NT Fisheries), Queensland (JCU), Indonesia (CSIRO* and RCFMC^#^), Papua New Guinea (NFA)	Genetic data	Population connectivity

*CSIRO: Commonwealth Scientific and Industrial Research Organisation (Australia); ^#^RCFMC: Research Centre for Fisheries Management and Conservation (Indonesia). Note that fisheries data for PNG are aggregated at a national level, and location data for Indonesian catch data were unavailable.

**Table 3:** Description and assessment of conceptual models developed to explain observed patterns of distribution and population structure of scalloped hammerhead sharks in the Assessment Region.

Hypothesis	Description	Current support	Future research results that would support hypothesis
Model 1: Panmictic population throughout region	Adults move freely through the region; adult females likely to return to natal nursery areas in northern Australia, PNG and Indonesia to give birth.	Genetic connection between Australia and Indonesia^48^,^50^; size and sex structure data (Fig. 3); ability to travel across deep water (from other regions)^30^,^71^.	Genetic analysis
		Moderate support	Tests comparing Australian, Indonesian, PNG and Pacific island samples show no differences with any type of marker (mtDNA, microsatellites, SNPs).
			Telemetry and tagging
			Tracking results of adults show movements from Australian waters into Indonesian and PNG waters
Model 2: Limited movement	Adults remain in restricted geographic areas (e.g. adults from Queensland coast move offshore to edge of shelf or Coral Sea Reefs) but rarely move to other areas.	Limited current support, contradicts genetic data.	Genetic analysis
		Limited support	Tests comparing Australian, Indonesian, PNG and Pacific island samples show significant differences between regions (possibly including within Australia) with any type of marker (mtDNA, microsatellites, SNPs).
			Telemetry and tagging
			Tracking results shows movement of adults to offshore areas but no long distance movements between countries.
			Fishing or diver surveys
			Sampling Australian shelf edge habitats and offshore seamounts identifies significant populations of adults (especially pregnant females).
Model 3: Continental shelf movement	Adults move along the margins of continental shelves, including northwards from Australia into eastern Indonesia (eastern Banda Sea) and PNG.	Genetic connection to Indonesia^48^; size and sex structure data; evidence of residency to continental shelves in other regions; ability to move large distances.	Genetic analysis
		Moderate support	Tests show connectivity between Australian samples and eastern Indonesia (eastern Banda Sea and West Papua) and PNG, but not western Indonesia and Pacific Islands.
			Telemetry and tagging
			Tracking results shows movements along continental shelves, but not across deep water.
Model 4: East-West Australian stock divide and continental shelf movements	Similar to the previous hypothesis but Torres Strait land bridge divides stocks to the east and west, with adults moving northwards into Indonesia (from WA, NT) or PNG (from Qld).	Similar to previous hypothesis; Torres Straight Land Bridge has caused population structuring in other sharks and teleost species^49^.	Genetic analysis.
		Moderate support	Tests show (1) connectivity between eastern Indonesia (eastern Banda Sea and West Papua) from NT and WA only, and PNG from eastern Queensland only; (2) no genetic connectivity with western Indonesia and Pacific Islands; 3) no genetic connectivity between eastern Queensland and the rest of northern Australia.
			Telemetry and tagging
			Tracking results shows movements along continental shelves but not through Torres Strait Land Bridge or across deep water.
